# State of household need for caregivers and determinants of psychological burden among caregivers of older people in Thailand: An analysis from national surveys on older persons

**DOI:** 10.1371/journal.pone.0226330

**Published:** 2019-12-11

**Authors:** Ruttana Phetsitong, Patama Vapattanawong, Malee Sunpuwan, Marc Völker

**Affiliations:** 1 Institute for Population and Social Research, Mahidol University, Nakhon Pathom, Thailand; 2 Faculty of Physical Therapy, Mahidol University, Nakhon Pathom, Thailand; Institute of Economic Growth, INDIA

## Abstract

**Objectives:**

To explore the levels and trends of household need for caregivers of older people and to explore the impact of potential determinants of psychological burden among caregivers of older people in Thailand.

**Methods:**

The secondary data analysis was performed using the Survey of Older Persons in Thailand 2007, 2011, and 2014 datasets which conducted by the National Statistical Office of Thailand. The household need for caregivers of older persons refers to having at least one older person in the household who needs a caregiver for assistance with activities of daily living. Caregiver mental health, which is confined to the available 2011 data, is defined as a state of psychological burden. Ordinal logistic regression models were used to explore the impact of potential determinants of psychological caregiver burden.

**Results:**

The household need for caregivers in Thailand tends to be increasing, from 5.0% in 2007 to 6.6% in 2014. The levels of the household need for caregivers were significantly higher in urban areas, Bangkok, and high socioeconomic households. In terms of psychological caregiver burden, the Thai Mental Health Indicators in 2011 produced, on average, a fair level of mental health, but one-fourth of caregivers had poor mental health. Duration of care for older people, household wealth, and functional dependency significantly predict total caregiver burden. Household characteristics are vital in predicting psychological caregiver burden, and the adjusted effect of the fifth quintile of household wealth was high (odds ratio = 2.34; 95% confidence interval = 1.47–3.73).

**Conclusion:**

The increasing need for caregiving in households with an older person can lead to a higher caregiver burden, particularly among those caregivers who care for dependent older people in poor households. Longer duration of caregiving is a factor that mitigates this burden.

## Introduction

Since the late 1980s, Thailand has been undergoing an epidemiological transition from communicable to non-communicable diseases. During this transition, which has been accompanied by demographic aging, more than 70% of Thai older people have had chronic illnesses, and 9.8% have been disabled [1]. Due to these impairments, older persons have had an increasing need for care services. To date, the health care system and social support from both the government and private sectors have not yet sufficiently responded to the need for caregiving toward the older population. Thus, Thai older people mainly rely on their family and community for care [2]. In the same household, individual Thai caregivers often need to take care of more than one older family member, a burden that can negatively affect their physical and psychological health, social life, as well as finances of the caregivers. The psychological caregiver burden has been identified as a serious health risk and mortality factor for both caregivers themselves and the older people they care for [3,4].

Several factors could influence caregiver’s mental health, including demographic characteristics (e.g., age-sex, race, ethnicity, geographic location) and social determinants (e.g., interpersonal, social class, socioeconomic status, housing quality, social support, and community dynamics) [5,6]. It has been well documented that the caregiver’s mental health had been adversely affected by caregiving [7,8]. Caregiving refers to activities and experiences involved in providing help and assistance to ordinary relationships or friends who are unable to provide for themselves with the activities of their daily living [9]. Compared to non-caregivers, caregivers have a higher risk of psychological burden [10,11].

Various factors in the process of caregiving that can affect caregiver burden, according to the Pearlin Stress Process [9] and the Stress Appraisal model of Yates [12], have been taken into account in empirical studies of caregiver burden. Guided by these two models, background and contextual (e.g., socioeconomic characteristics, caregiving history), stressors (e.g., functional dependency, cognitive impairment and behavioral problem of the older people, or economic problem of the caregiver), appraisal of the caregiving situation, and social support play a significant role in explaining variation of caregivers’ mental health. Factors that have been mentioned to be associated with the caregiver’s mental health can be divided into two main categories; (i) caregiver background and context characteristics and (ii) older people characteristics.

Background characteristics of the caregiver that may influence a caregivers’ psychological burden included the demographic and socioeconomic characteristics of the caregiver, relationship to older people. The context of caregiving may include the duration of care and the number of older people that caregiver provided care for them. Concerning the impact of caregivers’ demographic characteristics, such as age and sex, on caregiver burden, earlier studies have reported mixed results [13,14]. However, no gender difference was found in the level of caregiver burden, which could have been caused by contrasts in kinship roles and perceptions of caregiver burden, as found in previous longitudinal studies [15,16]. Caregivers with a relatively low socioeconomic status can be assumed to report a higher burden [17]. Less consistent findings were reported for the association between employment status and education level of the caregiver and their mental health. Concerning caregiver status, full-time caregivers experience worse mental health compared to part-time caregivers [18]. Moreover, the physical health status of caregivers (such as morbidity) is a factor that might contribute to their mental health. It has been evident that caregivers experiencing loss of physical strength tended to report poor mental health [19].

Regarding older people’s characteristics, caregiver burden is influenced by the level of physical health impairments of older people, which affects the degree of dependency and the required time and duration of care [20]. A recent meta-analysis has shown that functional dependency is highly correlated with the level of caregiver burden [21]. Beyond such individual factors, the impact of household characteristics, including household arrangement and socioeconomic status, on caregiver burden, has not yet been investigated.

In light of concerns about the accelerating need for long-term care and security of Thai older people, it is essential to have a thorough understanding of the household need for caregivers and caregiver burden. Understanding the bigger picture of the situation at the household level can provide answers to a range of data needs that can inform development and monitoring policies. Particularly in the case of Thailand, the decision to develop effective and sustainable long-term care for older people should be made by considering household characteristics, as well as considering the burden perceived by caregivers themselves, who are usually household members. As existing studies have been limited to individual-level data, there is a lack of evidence on how household factors affect caregiver burden. Therefore, the present study assesses the need for caregivers of older persons across time and explores the level and determinants of caregiver burden at the household level.

## Methods

### Data sources and sample

Data were obtained from the Survey of Older Persons in Thailand (SOPT) conducted in 2007, 2011, and 2014 by the National Statistical Office (NSO), Ministry of Information and Communication Technology of Thailand. The surveys were designed using a stratified two-stage sampling approach. The 76 and 77 provinces of Thailand were assigned as 76 and 77 strata in the 2007 survey, and the 2011 and 2014 surveys, respectively. Each stratum was divided into two administrative areas called in- and out-municipality areas. The primary sampling units were blocks for in-municipality areas and villages for out-municipality areas. The selection probabilities were proportional to the size of the number of dwelling units in a block or village. The secondary sampling units were private households using systematic random sampling from a list of all households in the selected block or village. Fifteen private households per block and twelve private households per village were selected in the 2007 and 2011 surveys, whereas sixteen households per block and twelve households per village were selected in the 2014 survey. A structured questionnaire was used to collect both household and individual data from the respondents. All persons aged 50 years or older in each sample household were interviewed for older-person-related questions (e.g., health status and health behavior, preparation for the old age of older persons, participation in activities). Besides, the Mental Health Survey (the Thai Mental Health Indicator) for older persons aged 60 years or older, and their caregivers were included in the SOPT 2011. Notably, for the household and individual datasets of the SOPT 2011 and 2014, the data from all sample households are allowed for public use. A sub-set of only households with a person aged 50 years or older can be obtained from the SOPT 2007.

The present study focused on those households with people aged 60 years or older, as it is the standard age threshold to define ‘older’ persons in Thailand. All households with older people were included in the analysis of the household need for caregivers. After data quality assessment by systematic data editing and cleaning, the final representative sample included 22 688 households in 2007, 25 077 households in 2011, and 27 990 households in 2014. In the analysis of caregiver burden, the study included only households with older people’s primary caregiver whose mental health status was assessed.

### Measures

#### Household need for caregivers of older persons

The household need for caregivers of older persons is defined as having at least one older person in the household who needs a caregiver for caregiving. It was assessed based on an older person’s difficulty with activities of daily living (ADLs). According to relevant recent literature, the difficulties associated with standard basic ADLs that suggest a need for caregivers to provide day-to-day care include eating, dressing, bathing, and using the toilet [22,23]. In SOPT 2007, 2011, and 2014, older persons were asked, “*Can you perform the activities of daily living (eating*, *dressing*, *bathing*, *toileting*, *and brushing your teeth and cleansing your face)*?” The performance levels ranged from “*unable*” to “*need some help*, *but can do some things alone*” to “*independent*.” In this study, older persons who answered that they are “*unable*” or that they “*need some help*, *but can do some things alone*” with regards to one or more of the five ADLs were classified as “*in need for caregivers*.” The wording of the questions is identical across the survey, but the response codes in the SOPT 2007 are different from the SOPT 2011 and 2014. Therefore, data were recoded for harmonizing.

#### Psychological caregiver burden

Focusing on the psychological caregiver burden, only the SOPT 2011 had questions on the mental health of the caregiver. The Thai Mental Health Indicator (TMHI-15), developed by the Department of Mental Health of the Ministry of Public Health (MOPH), was adopted to assess the caregiver’s mental health. It is a self-assessed measurement which has been developed into a construct valid and reliable instrument for assessing the mental health of the population within the context of Thai society and culture [24,25]. The TMHI has been used to investigate the national happiness of Thai people in almost every year since 2001 [26] and is commonly used in the research on mental health among the Thais [18,27,28]. The data from this tool can help inform policy to promote and prevent the mental health of the Thai people [25].

The 15-items TMHI-15 consists of four domains: mental state, mental capacity, mental quality, supporting factors. The first domain (the mental state) includes both positive mental state with three items (satisfied with life, relaxed, and good self-esteem) and negative mental state with three items (feel bored, feel disappointed, and feel unhappy with life). The second domain (mental capacity) consists of three items (can accept problems, can control emotions, and confident in facing bad situations). The third domain (mental quality) comprises three items (sympathetic towards others, happy in helping others, and help others when to have the chance). The last domain (the social support) includes three items (feel secure and safe with family, believe that family will take care of when the ill, family members love and care for each other). The questions concern psychological experiences during the month prior to the survey. The measurement scale of each item included the following levels: 0 = never; 1 = a little; 2 = a lot; and 3 = very much. The norm values of TMHI-15 were classified into three standard groups; poor (≤27), fair (27.01–34), and good mental health (>34) [24]. The full questionnaire is available in the S1 File.

#### Determinants of psychological caregiver burden

To capture possible determinants of caregiver burden, the determinants including sex, age group, education level, relationship to the older person, duration of care for the older person, care for more than one older person, relative household wealth, household arrangements by age group of the older person, and functional dependency of the older person are our concerns.

The caregiver’s sex refers to the sex of the primary caregiver at the time of the survey. It has been suggested that, in Thailand, there is socio-cultural pressure on females to adopt the role of family caregiver. However, women have difficulty balancing their family roles and job responsibilities, and that makes the experience stressful [29]. This difficulty has led to the view that there may be differences between male and female caregivers on caregiving outcomes such as mental health of the caregiver.

The caregiver’s age is the age of the primary caregiver at the time of the survey. The difference in age of caregivers affects their mental health due to related stress and morbidity [30]. The older caregivers tend to suffer from mental health problems more than non-older caregivers. In this study, two age groups of the caregivers were categorized according to standard age range used to define older people in Thailand; older caregivers (who aged 60 years or over), vs. non-older caregivers (who aged 59 years or under).

The caregiver’s education level refers to highest competed level of education of the primary caregiver at the time of the survey. Education level is used as the indicator of socioeconomic status of the caregivers, and it is one of the key factors affecting mental health. Self-esteem has been found to be positively correlated with caregiver's level of education [19]. In this study, the level of education of the caregivers was divided into three categories; primary or less, secondary, and higher than secondary.

The relationship of the caregiver to the older persons is the relationship of the primary caregiver to the older at the time of the survey. Kinship is another important caregiver demographic characteristics to consider in determining the caregiver burden [16]. Kinship may intensify the potential effects of family caregiving [31,32]. A caregiver might provide quality physical care for older people in the household, but perceptions of familial responsibilities in caregiving might increase feelings of subjective burden [33]. In this study, kinship status of the caregivers refers to having a family tie (i.e., being a spouse, child, child-in-law, grandchild, sibling, parents) with the older persons, whereas non-kinship caregivers have no family tie (i.e., are an employee/servant, nurse, assistant nurse, and paid caregiver).

The duration of care for older persons is the duration of being a caregiver at the time of the survey. The more confining the caregiving task, the more likely that it will create adverse effects on mental health. The duration was measured using the questions asked of the primary caregivers as “*How long have you given care to the older person*? *(All older persons you ever gave care to)*”. The duration of care was categorized into three groups; two years or less, three to four years, and more than four years [13,34].

Care for more than one older person refers to a double burden status of caregivers in caregiving. The larger the number of older household members who need caregiving, the more likely that the caregivers will suffer adverse mental health consequences. This variable was obtained from the data regarding the number of older persons who need for caregivers as measured by ADL indicator in the same household.

A relative household wealth of older persons is a proxy of the socioeconomic status of the older person’s household. It was measured based on a set of household characteristics and assets ownership. These attributes were converted into a wealth index by principal components analysis (PCA) [35] and assigned the range of a wealth index score to classify households by wealth quintile (1 = poorest, 2 = poor, 3 = medium, 4 = rich, and 5 = richest). The relative household wealth of the older person’s households was derived from the construction of the wealth index of all sample households in the SOPT 2011 and 2014. For the SOPT 2007, only data from households with persons age 50 years or over are publicly accessible. Therefore, the household wealth variable could not be used for the year 2007.

Household arrangement by older person’s age group refers to the household composition of older persons according to the number and age groups (young level = 60–69 years, middle level = 70–79 years, and oldest level = 80 years or over) of older household members at the time of the survey. At the household level, the presence of multiple older members (more than one older person) is the norm. Caregiving for more than one older household member of different ages might create an additional need for caregivers, and the caregiver might be caring for older people with considerably different levels of needs. To analyse the determinants of the psychological caregiver burden, the sample households were classified into four types: (I) households with one young-level or one middle-level older person, (II) households with one oldest-level older person, (III) households with more than one older person, with young-level and/or one middle-level older person, and (IV) households with more than one older person, and with at least one oldest-level older person.

To investigate factors affecting psychological caregiver burden, household arrangement by older person’s functional dependency refers to the household composition of older persons according to the functional dependency of the older household members at the time of the survey. The functional dependency of older people is a significant variable to the level of the mental health burden experiencing by the caregivers [36,37]. Also, the sample households were classified into two types by considering the functional dependency of older household members: (I) the households with all independent older people, (II) the household with at least one dependent older person. The level of functional dependency was determined based on assessing ten ADLs measured by the Barthel Index Scale [38]. These ADLs include feeding, grooming, transferring, toilet use, mobility, dressing, stairs, bathing, bowels, and bladder. However, there is no question related to transferring in the SOPT 2011. The measurement scale of each ability to perform the activities included the following levels: “0 = no”, “1 = yes, with aid” “2 = yes, without aid”. The cut-off scores were determined corresponding to the guidance of the Institute of Geriatric Medicine, Thailand (≥10 = independency; 4–9 = partial dependency; and ≤3 = dependency out of 18 points).

### Analytical approach

Data analysis was conducted at the household level using STATA/SE 14.0 (StataCorp, Texas, USA). Descriptive analysis was used to characterize the sample, to examine the distribution properties of the variables, and to explore the level of household need for caregivers of older persons. Before data analysis, a complex sample plan (csplan) was applied in the descriptive analysis. Ordinal logistic regression were used to explore the impact of potential determinants of psychological caregiver burden. It appropriates the ordinal nature of the data by allowing for more than two (ordered) response categories of the outcome variable without the need for an exact distance between them [39,40].

Ethical approval for this study was obtained from the Institute for Population and Social Research-Institutional Review Board (IPSR-IRB) on 29 June 2017 (COA No. 2017/06-135).

## Results

### Household need for caregivers of older persons

The percentage of households in need for caregivers of older people varied between 2007 and 2014. In 2007, 5.0% of all households were in need for caregivers. In 2011, this percentage was slightly lower (4.6%). The percentage was, however, higher in 2014 (6.6%).

Differentiated by area of residence, the percentage of household need for a caregiver in urban areas decreased from 6.8% in 2007 to 5.8% in 2011, and then returned to the same level as in 2007 (6.8%). Likewise, the proportion of household need for caregivers in rural areas declined from 4.3% in 2007 to 4.0% in 2011, and then rapidly increased to 6.4% in 2014. The findings clearly show that the level of household need for caregivers of older persons in 2007 and 2011 was significantly higher in urban areas. However, with the rapidly increasing need for caregivers since 2011 in rural areas, the rural-urban differential disappeared by 2014 (Table 1).

**Table 1:** Percentage of household need for caregivers of older persons by area of residence, region, relative household wealth, and educational attainment of household head, 2007, 2011, and 2014.

Characteristics	2007	2011	2014
**Overall**	**5.0**	**4.6**	**6.6**
**Area of residence**			
Urban	6.8	5.8	6.8
Rural	4.3	4.0	6.4
	*p<0*.*001*	*p<0*.*001*	*p = 0*.*404*
Design-based test (adjusted F)	24.699	21.421	0.697
**Region**			
Bangkok	8.4	7.8	9.8
Central (excluding Bangkok)	5.9	5.9	7.4
Northern	4.1	4.2	4.2
Northeastern	4.0	3.0	6.2
Southern	5.5	4.9	7.2
	*p<0*.*001*	*p<0*.*001*	*p<0*.*001*
Design-based test (adjusted F)	8.641	16.748	14.538
**Relative household wealth**			
Quintile 1, poorest	na.	4.1	6.1
Quintile 2, poor	na.	3.7	5.9
Quintile 3, medium	na.	4.5	7.0
Quintile 4, rich	na.	4.7	7.2
Quintile 5, richest	na.	6.3	7.2
		*p<0*.*001*	*p = 0*.*063*
Design-based test (adjusted F)		5.456	2.269
**Educational attainment of household head**			
Primary or less	na.	4.4	6.5
Secondary	na.	5.0	6.5
Higher than secondary	na.	7.0	7.6
		*p<0*.*01*	*p = 0*.*181*
Design-based test (adjusted F)		5.791	1.711

Note: calculated from weighted samples under complex samples analysis

Concerning region of Thailand, regional terms (Bangkok, Central [excluding Bangkok], North, Northeast, and South) defined by the Thai government reflect geographical and cultural variations of the country. Bangkok had the highest percentage of household need for caregivers of older people in 2007, 2011, and 2014 (8.4%, 7.8%, and 9.8%, respectively). The lowest percentage (approximately 4%) was found in the North region. During 2007, 2011 to 2014, the household need for caregivers of older persons in the Central region were increasing, while need in the North was relatively stable. Fluctuating trends were found in Bangkok, the Northeast, and the Southern (Table 1).

When taking household socioeconomic status using the household wealth index into consideration, there was a significant difference in the household need for caregivers across household wealth in 2011 (*p*<0.001). The finding showed that the prevalence of the household need for caregivers was highest in the richest households (6.3% in 2011, and 7.2% in 2014). On the other hand, the poor households presented the lowest percentage of the need for caregivers, accounting for 3.7% and 5.9% in 2011 and 2014, respectively (Table 1).

In terms of educational attainment of household head, the household need for caregivers of older persons was highest among the households where household head had completed higher than secondary school (7.0% in 2011, and 7.6% in 2014), compare to those where household head had highest educational level of secondary school (5.0% in 2011, and 6.5% in 2014) and primary or less (4.4% in 2011, and 6.5% in 2014), though the difference was not statistically significant in 2014 (Table 1).

It is noted that the prevalence of household need for caregivers of older persons by relative household wealth and educational attainment of household head in 2007 could not be calculated since the data on total households were not publicly accessible as described in the Methods section (Table 1).

### Psychological caregiver burden

Out of 25 077 older person households in the 2011 survey sample, 642 households with older household members who had caregivers were included in the psychological caregiver burden analysis. A total of 1 102 older persons lived in those 642 households, with a mean age of 75.8 years (SD = 9.8; range = 60–117). The primary caregiver’s average age was 49.5 years (SD = 12.8; range = 15–84), and a vast majority of caregivers were women (83.5%) and had family ties to the older person in need of caregiving (93.6%). Nearly two-thirds of caregivers had an educational level of primary school or less. Most caregivers had provided care for the older person for more than four years (71.0%) (Table 2).

**Table 2:** Percentage distribution of mental health status of caregiver by caregiver characteristics, household characteristics and household arrangement, 2011.

Characteristics	Category	Caregiver Mental Health Status (%)			Total (n)
		Poor	Fair	Good	
Overall			25.4	51.6	23.0	642
***Caregiver characteristics***						
Sex	Male		25.5	49.1	25.5	106
	Female		25.4	52.1	22.6	536
Age group	Non-older		24.3	51.7	23.9	518
	Older		29.8	50.8	19.4	124
Education level	Primary or less		29.9	50.6	19.5	401
	Secondary		21.0	50.0	29.0	124
	Higher than secondary		14.5	56.4	29.1	117
Relationship to the older person	Kinship		26.3	50.4	23.3	601
	Non-kinship		12.2	68.3	19.5	41
Duration of care for an older person	≤2 years		31.5	54.6	13.9	108
	3–4 years		25.6	55.1	19.2	78
	>4 years		23.9	50.2	25.9	456
Care for >1 older person	No		25.4	51.9	22.7	582
	Yes		25.0	48.3	26.7	60
***Household characteristics***						
Relative household wealth	Quintile 1, poorest		41.0	45.8	13.2	144
	Quintile 2		27.6	51.4	21.0	105
	Quintile 3		23.6	50.0	26.4	106
	Quintile 4		18.8	57.3	24.0	96
	Quintile 5, richest		16.8	53.9	29.3	191
***Household arrangement by older people characteristics***						
By age group	Type I: households with 1 young-level or 1 middle-level older person		21.8	52.4	25.7	206
	Type II: households with one oldest-level older person		23.5	53.6	23.0	196
	Type III: households with >1 older person with young-level and/or middle-level older person		25.2	50.5	24.3	111
	Type IV: households with >1 older people, and with ≥ 1 oldest-level older person		34.1	48.1	17.8	129
By functional dependency	All independent older people		19.8	52.3	27.9	394
	At least 1 dependent older person		34.3	50.4	15.3	248

### Level of caregivers’ mental health

The results showed that the mean mental health score of caregivers was 30.9 out of a total score of 45. As summarised, scores are interpreted into three categories; poor (≤27), fair (27.01–34), and good (>34). Applying these categories to our context of self-perceived psychological caregiver burden suggests that the majority of caregivers have the perception of having a fair level of mental health (51.6%). However, 23.0% of the caregivers perceived their mental health to be good, while 25.4% perceived their mental health to be poor. Table 2 breaks down the socio-demographic characteristics of the primary caregivers and summarises the caregiver characteristics, the relative household wealth, and the household arrangement by the older people’s characteristics, including age group and functional dependency.

### The impact of potential determinants of the mental health of caregivers of Thai older people

In order to investigate the factors affecting the caregiver’s mental health (ordinal scale with 1 = poor, 2 = fair, and 3 = good), ordinal logistic regression was employed. According to an insignificant Brant test of the parallel line, the proportional odds assumption is not violated in our data. Therefore, the results of the ordinal logistic regression of caregivers’ mental health status are presented in Table 3. Interpretation of the ordinal logistic regression results is a comparison among the odds of having poor mental health versus having combined fair and good mental health, and the odds of having combined poor and fair mental health versus having good mental health.

**Table 3:** Odds ratio (OR) and 95% confidence interval (CI) from ordinal logistic models predicting mental health status among caregivers of older people in households (n = 642 households), 2011.

Selected Predictors	Model 1		Model 2		Model 3	
	OR	95%CI	OR	95%CI	OR	95%CI
***Caregiver characteristics***						
Sex (ref: Male)						
Female	1.01	0.67, 1.50	0.97	0.64, 1.46	1.05	0.70, 1.58
Age group (ref: Non-older)						
Older	0.87	0.60, 1.28	0.94	0.63, 1.38	1.09	0.67, 1.78
Education level (ref: Primary or less)						
Secondary	1.63*	1.09, 2.43	1.34	0.89, 2.03	1.33	0.88, 2.01
Higher than secondary	1.95***	1.31, 2.90	1.51	0.97, 2.33	1.42	0.91, 2.21
Relationship to older people (ref: Kinship)						
Non-kinship	1.52	0.84, 2.75	1.35	0.74, 2.46	1.51	0.82, 2.77
Duration of care for older people (ref; ≤2 years)						
3–4 years	1.46	0.84, 2.53	1.47	0.83, 2.58	1.39	0.78, 2.45
>4 years	1.82**	1.22, 2.74	1.79**	1.19, 2.70	1.70*	1.12, 2.57
Care for >1 older people (ref: No)						
Yes	1.03	0.62, 1.73	1.02	0.61, 1.72	1.11	0.60, 2.04
***Household characteristics***						
Household wealth (ref: Quintile 1, poorest)						
Quintile 2			1.79*	1.09, 2.94	1.60	0.96, 2.66
Quintile 3			2.21**	1.34, 3.63	2.09**	1.26, 3.46
Quintile 4			2.27**	1.37, 3.75	2.20**	1.32, 3.67
Quintile 5 (richest)			2.53***	1.60, 4.02	2.34***	1.47, 3.73
***Household arrangement by older people characteristics***						
By age group (ref: Type I: households with 1 young or 1 middle-level older person)						
Type II: households with 1 oldest level older person					1.04	0.71, 1.53
Type III: households with >1 older person with young and/or middle level older person					0.95	0.52, 1.60
Type IV: households with >1 older people, and with at least 1 oldest level older person					0.62	037, 1.04
By functional dependency (ref: All independent older people)						
At least 1 dependent older person					0.53***	0.39, 0.74
Log likelihood	-647.347		-637.994		-627.327	
LR chi^2^	25.10		43.80		65.14	
Prob > chi^2^	0.0015		0.0003		0.0000	
Pseudo R^2^	0.0190		0.3250		0.0494	
AIC	1315		1304		1291	

Note: Dependent variables measure caregivers’ mental health (ordinal scale with 1 = poor, 2 = fair, and 3 = good)

*** P<0.001;

**P<0.01; and

*P<0.05

A hierarchical multiple regression approach was employed to examine the relationship of a set of predictors on caregivers’ mental health status. In Model 1, only caregiver characteristics were taken into account. The results showed that educational level and duration of care for older people had a significant effect on caregivers’ mental health. Caregivers with secondary or higher-than-secondary education were more likely to report a better mental health status than caregivers with primary or less schooling. Caregivers with secondary or higher-than-secondary education had better mental health than those with only primary or less education, by 1.63 times and almost two times, respectively. In other words, lower educational attainment was associated with caregiver burden. The odds of having reported a better mental health status were 82% higher for caregivers who had provided care to the older person for more than four years compared to those who had less duration of care.

In Model 2, which included both variables measuring caregiver and household characteristics, the relative wealth of households is an additional significant correlate of caregiver’s mental health. Caregivers in the poorest quintile of households were more likely than others to report a low mental health status. Moreover, the effect of education on a caregiver’s mental health became insignificant. While the duration of care remained statistically significant, the size of the odds ratio was reduced.

Finally, Model 3, which also included a household arrangement in terms of older household member characteristics, indicates that having at least one functional-dependent older person in the household led to a worsening of mental health among caregivers by 47%. No significant association could be identified between the age structure of older household members and caregiver’s mental health. It is noteworthy that Model 3 shows that caregiver’s education level was no longer a significant predictor of mental health, although the duration of care and relative household wealth still were. In other words, the duration of care for older people and household wealth significantly predicted the caregiver’s overall mental health, even when a primary stressor, such as the older people’s functional dependency, was controlled.

While, according to the Pseudo R squared, Model 2 is preferred, the Akaike’s Information Criterion (AIC) indicates that Model 3 is the superior model. Notably, AIC takes into account both a model’s goodness-of-fit and its simplicity in terms of the number of parameters needed to achieve this fit.

## Discussion

The average number of older persons, as well as the proportion of older persons in Thai households, has increased in recent years, while the proportion of older person households has risen slightly from 2007 to 2014. As a result, age-related morbidity and disability have been on the rise [30]. Difficulty performing ADLs is evidence of a disability. The present study investigated basic ADLs performance among older household members as an indicator of the household need for caregivers of older persons, and it found that the percentage of households in need for caregivers of the older persons slightly declined, from 5.0% to 4.6%, between 2007 and 2011, and then increased to 6.6% in 2014. Despite these small percentages, an increasing trend of households in need for caregivers of older persons has been observed. The household need for caregivers in the period between 2007 and 2011 was relatively stable, with only minor fluctuations. It may be explained by the fact that more Thai older people in 2011 had received a physical check-up than those in 2007, and a higher proportion of the older people were reported to be in very good health in 2011 [41]. In the year 2014, the household need for caregivers increased, and that could reflect the long-term trend of a growing proportion of older persons in Thai households. This finding presents a challenge because care for older people in Thailand is still mainly provided by family and community members. Moreover, households with older persons face higher levels of health expenditures compared to households without older people [42]. These challenges are exacerbated by the fact that more than half of the Thai population has no retirement pension [43].

Variations in household need for caregivers of older people can be clarified by area of residence, region, household wealth, and household head’s education level. Socioeconomic behaviors and cultural bias may be manifest in different patterns of lifestyle, health-related preferences, health-seeking behavior, as well as perceptions of older people, which varies depending on area of residence and region [44].

The comparison of household needs for caregivers of older persons between urban and rural areas shows that the prevalence of household needs for caregivers was higher in urban areas over time. It is possible that living in a city is more harmful to health as a person ages. Some studies found that higher urbanization rates were associated with environment-related morbidity [45,46]. Air pollution is a major cause of pulmonary disease and cognitive impairment among older persons. Additional, the higher population density in cities means that there is usually an inadequacy of outdoor areas for exercise and recreation. By contrast, various social aspects of rural life promote positive health outcomes, such as strong social networks, norms of neighborliness, a greater emphasis on qualitative aspects of life, and norms of self-help and reciprocity [47].

However, it is crucial to not overlook the sharp increase in the need for caregivers in older-person households in rural areas; there was only a small percentage difference in the household need for caregivers between rural and urban areas as of 2014. These findings might reflect the demographic and socioeconomic shifts in rural areas of Thailand, which are becoming more modern. Socioeconomic improvement influences lifestyle choices that are associated with health behaviors of people in that area [48]. Rural communities are increasingly adopting urban values and ways of life. One study found that the pattern of chronic diseases of older people in rural and urban areas was not that different; the prevalence of hypertension, obesity, and physical inactivity of older-person households in rural areas were almost the same as in urban areas [49].

Significant regional differences in household need for caregivers of older persons were evident in this study. By 2000, Bangkok accounted for half of the country’s urban population. The quality of health of the urban population is influenced by economic growth, which has led to changes in culture, society, and the environment. Therefore, it is not surprising that Bangkok ranked highest for household needs for caregivers of older persons during 2007, 2011, and 2014. Lifestyle factors and health behavior (e.g., alcohol use and smoking) differ by region; it has been reported that there was higher prevalence of smoking by older people in Bangkok and the Central region [50].

Moreover, the previous study revealed that older persons in Bangkok had the lowest active aging index amongst the five regions [51]. The North had the highest percentage of older-person households (of total households), yet also had the lowest prevalence of household need for caregivers of older persons, and the trend was relatively stable. That finding might partly be explained by more engagement in social activities and self-reliance of older people in the North [52]. Indeed, further analyses on the data for this study found that the prevalence of being a member and actively participating in old age citizen’s groups in the North region was higher than all the other regions and across time.

By concerning socioeconomic inequality of older-person households in Thailand, differences in household need for caregivers of older persons by their household wealth and household head’s education should be considered.

This study highlighted the equality profile of household needs for caregivers of older people by household wealth. In 2011, the household need for caregivers was highest among the richest households, while poor socioeconomic status presented the lowest percentage. Although the relationship between socioeconomic status and health has been well documented, less evidence has been presented to support our findings. Older members households with highest socioeconomic status had poorer health, resulting in higher need for caregivers. One explanation is that, in 2011, the proportion of households with at least one oldest-old person was highest in the richest households, and was lowest in the poor socioeconomic households. Having household members age 80 years or over (oldest-old age group) is a significant driver of need for caregivers of the household. It stands to reason that the oldest-old age group has higher prevalence of disease and disability, including dementia [53].

Moreover, the findings of this study pointed out that, in 2011, educational attainment of the household head was positively correlated with the household need for caregivers. Households with higher education level of household head had a higher need for caregivers of the older household members compared to lower educated-headed ones. Indeed, the high educated-headed households in this study were more likely than others to be households with a high number of older household members. Other things being equal, the higher the number of older persons in a household, the higher the household need for caregivers.

These findings suggested that compared to older people of low socioeconomic households, those of high socioeconomic households lived longer but with a disability as measured with ADLs.

In terms of the resulting burden on caregivers, this study found that, for most caregivers, the psychological burden, as defined by the average TMHI-15, was relatively low. Thai caregivers experienced both positive and negative effects on their mental health. Supporting with a previous study indicated that the psychological burden among Thai caregivers is low compared to other dimensions of burden [13]. However, under- or over-reporting of self-rated mental health problems could have happened depending on the relationship’s quality, the attitude towards the care, and cultural norm and prescriptive. In Asian culture, there is a great sense of obligation for the care of older family members, and that might have biased self-reporting toward comparatively low psychological burden. Moreover, most Thai people are Buddhist, which might also lead to the under-reporting of mental health problems as the Buddhist concept of searching for a path of moderation between two extremes is frequently applied to caregiving [54,55]. Other possible explanations, such as the provision of care by other family members, by paid care, or by village volunteers in the local community, might be reasons for the low burden.

Despite the relatively low overall psychological burden of caregiving, about one-quarter of the sample of caregivers in this study indicated a substantial mental burden of caregiving. It is, therefore, essential to study the determinants of their burden in more detail. The risk factors associated with caregiver burden in this study can be classified into individual caregiver factors and household factors, including household socioeconomic status and household arrangements by older people factors.

Another factor related to caregiver burden is the duration of care. A majority (71%) of Thai caregivers have been looking after their older persons for more than four years. The duration of care of older people affects the caregiver burden. Interestingly, a longer duration of care negatively impacts on the caregiver burden. Our results show that longer contact time with their older people predicts better mental health among caregivers. This phenomenon of duration affecting caregiver burden can be explained by the “*adaptive mechanism*” whereby the caregivers gain a better understanding of the conditions of their older people and acquires improved coping skills from the role of caregiving over time [34,56]. Similarly, a qualitative study of caregivers of stroke patients in a rural setting found that caregiving became more manageable over time [57]. Nevertheless, this finding contrasts with another study on burden of Asian caregivers of people with dementia, where the duration of care had no significant correlation with caregiver stress [58].

In our analysis, the wealth of the older people household was found to have the longest and strongest positive impact on mental health. It implies that household characteristics are crucial in predicting psychological caregiver burden. This finding adds essential insight to the existing empirical evidence on caregiver burden since few previous studies have simultaneously modeled individual- and household-level determinants of caregiver burden. The caregivers who provided caregiving for the older household members of high economic status were less likely to suffer from the psychological burden. One can hypothesize that wealthier households are more likely to allocate financial resources to pay for health care facilities and services for caregiving. However, while most existing studies have reported the economic consequences of caregiving and out-of-pocket spending for elder care of households, little is known about the impact of the household economic status on caregiver burden. One study by Prince *et al*. [59] in low- and middle-income countries suggested that there was no consistent evidence for an association between a household’s socioeconomic status and caregiver burden. The number of household assets was inversely associated with burden in some settings, such as Venezuela and rural Peru, with a strong but insignificant trend in the opposite direction in urban sites in India and China.

Consistent with the existing studies [60–62], our study has also found that caregiver burden was significantly associated with older people’s functional performance. The average TMHI-15 score was different from the previous reports [18] based on the severity of functional dependency or illness severity of the older person and demographic factors such as age. As age increases, functional ability generally decreases, and older persons tend to become more dependent on others in their ADLs.

Finally, the present study found no significant association between caregiver burden and other caregiver characteristics, including sex, age, kinship, number of older people to whom care was provided, and the household arrangement by the older people’s age group. The caregiver’s age-sex and kinship differences are frequently cited in the literature, and the results of this study are inconsistent with some previous studies [16,33,63–65]. There was no evidence for a significant effect of the number of older people to whom care was provided and the household arrangement by the older people’s age on caregiver burden. The impact of these characteristics on caregiver burden should be investigated further.

The strength of the present study is that it analyzed data from a national population-based study with large sample size. However, there are some limitations to the study. Firstly, the study is cross-sectional, which limits insight into the dynamics of caregiver burden over time. Secondly, the study was limited to private households. Therefore, the small number of older persons who were in institutional care or living in religious institutions were omitted. Thirdly, as standard household-based measures of need for caregivers in caregiving of older persons at the household level have been no available, this study investigated basic ADLs performance among older household members as an indicator of it. However, the indicator might not directly account for the psycho-social needs of older persons individuals. Lastly, the caregiver information in the survey has been limited. The caregiver characteristics included in the study were restricted to sociodemographic characteristics, the relationship of the caregiver to the older people, duration of care and caring for more than one older person. This study, however, has considered that some other potential caregiver factors such as occupational status and caregiving status (part-time/full-time) as well as health morbidities of the caregiver are also likely to be the determinants of mental burden of the caregivers, however, there were no available data on these variables in the surveys. Also, the interpretation of some parts of the results requires caution. The sample household weights of the SOPT carried out by the NSO were assigned to estimate the total number of households in the population. The sample weights of the SOPT 2007 and 2011 were calculated using data from the Population Projections for Thailand, 2000–2025, whereas the sample weight of the SOPT 2014 was computed from the Population Projections for Thailand, 2010–2040. Thus, the descriptive findings regarding the percentage of household need for caregivers between 2007, 2011, and 2014 are not directly comparable.

Beyond personal backgrounds, household characteristics have a profound effect on the situation of care for older people. In the case of Thailand, it is still unclear how current care provision mitigates the burden of caring. Suitable interventions to reduce caregiver burden should improve both caregivers and older person outcomes. Assessment of older people's and caregivers' needs and the consideration of related factors should be incorporated into treatment plans. Providing adequate funding for intensive programs and training at home or the local community should be provided to buffer the caregiver burden from the increasing number of older people households with care needs [66].

## Conclusion

The present study provides a fuller picture of the household need for caregivers of Thai older people and contributes to a better understanding of the factors that associate a psychological burden on their caregivers at the household level. Households with older people who live with ADLs difficulties and Thailand’s health care system as a whole will face challenges in meeting older persons’ care needs. In particular, when the proportion of older persons in Thai households is growing. The strong inverse relationship between household socioeconomic status of older persons and psychological caregiver burden was found in this study. However, social support, especially from within the household or the family, remains a crucial role for informal care of Thai older people. Psychological support from family, friends, and neighbors encourages caregivers to continue their care.

## Supporting information

S1 File Mental Health Survey (Self administered). (PDF)
