# Supplementary material for: State of household need for caregivers and determinants of psychological burden among caregivers of older people in Thailand: An analysis from national surveys on older persons
Source: PLoS One. 2019 Dec 11;14(12):e0226330. doi: 10.1371/journal.pone.0226330 (PMC6905555; doi:10.1371/journal.pone.0226330)
Supplement: S1 File — (PDF) [file pone.0226330.s001.pdf]

No.......of..........for this household

Mental Health Survey *(Self administered)*

(Ask older person aged 60 and over and care giver)

REG	CWT	AMP	TMB	AREA	ED	BLK_VIL	PSU_NO	MONTH_YR	HH_NO
1		4		8	9	12	14	18 5 4	22

The following questions concern your psychological experiences during past month

Record code "1 - 4" in ☐ according to your opinion or feeling.

No = 1 A little = 2 Much = 3 Very much = 4

REC_NO 6 34

• For persons aged 60 and over, record line no. and name from questionnaire SPS.54 (Long form) into A1 and A2	A1	32				
• For care giver, record line no. and name of the person whom the caregiver take care of and check "X" in "CG"	A2		name..................	name..................	name..................	name..................
	CG	35				
1. You are satisfied with your life	M01	36				
2. You feel relaxed/at ease..	M02	37				
3. You feel bored with your daily life	M03	38				
4. You feel disappointed in yourself	M04	39				
5. You feel that your life is unhappy..	M05	40				
6. You can accept a bad situation that is hard to be solved (when a problem occurs)..	M06	41				
7. You are confident you can control yourself when something bad or serious happens..	M07	42				
8. You are confident you can face bad situations in your life	M08	43				
9. You feel sympathy when seeing someone in trouble	M09	44				
10. You feel happy when helping someone in trouble	M10	45				
11. You help people when you have a chance	M11	46				
12. You feel proud of yourself	M12	47				
13. You feel secure and safe with your family	M13	48				
14. You believe that your family will take good care of you when you are seriously ill	M14	49				
15. Your family members love and care for each other........................	M15	50				

Source: The 2011 Surveys of Older Persons in Thailand

Available (in Thai) at: http://www.dmh.go.th/test/download/files/TMHI15_thai.pdf
